# Computational Analysis of Histamine Protonation Effects on H_1_R Binding

**DOI:** 10.3390/molecules28093774

**Published:** 2023-04-27

**Authors:** Marcus Conrad, Anselm H. C. Horn, Heinrich Sticht

**Affiliations:** 1 Division of Bioinformatics, Institute of Biochemistry, Friedrich-Alexander-Universität Erlangen-Nürnberg (FAU), 91054 Erlangen, Germany; mar.conrad@fau.de (M.C.); anselm.horn@fau.de (A.H.C.H.); 2 Erlangen National High Performance Computing Center (NHR@FAU), Friedrich-Alexander-Universität Erlangen-Nürnberg (FAU), 91058 Erlangen, Germany

**Keywords:** H_1_R, histamine, GPCR, tautomers, G_q_, protonation, molecular dynamics

## Abstract

Despite numerous studies investigating histamine and its receptors, the impact of histamine protonation states on binding to the histamine H${}_{1}$-receptor (H${}_{1}$R) has remained elusive. Therefore, we assessed the influence of different histamine tautomers (τ-tautomer, π-tautomer) and charge states (mono- vs. dicationic) on the interaction with the ternary histamine-H${}_{1}$R-G_q_ complex. In atomistic molecular dynamics simulations, the τ-tautomer formed stable interactions with the receptor, while the π-tautomer induced a rotation of the histamine ring by 180° and formed only weaker hydrogen bonding interactions. This suggests that the τ-tautomer is more relevant for stabilization of the active ternary histamine-H${}_{1}$R-G_q_ complex. In addition to the two monocationic tautomers, the binding of dicationic histamine was investigated, whose interaction with the H${}_{1}$R had been observed in a previous experimental study. Our simulations showed that the dication is less compatible with the ternary histamine-H${}_{1}$R-G_q_ complex and rather induces an inactive conformation in the absence of the G_q_ protein. Our data thus indicate that the charge state of histamine critically affects its interactions with the H${}_{1}$R. Ultimately these findings might have implications for the future development of new ligands that stabilize distinct H${}_{1}$R activation states.

## 1. Introduction

The histamine H${}_{1}$ receptor (H${}_{1}$R) is a G protein-coupled receptor (GPCR) that is expressed in many different cell types, including neurons, immune cells, vascular endothelial cells, and smooth muscle cells of the airway or intestinal epithelium [1]. The H${}_{1}$R plays an important role in type I hypersensitivity reactions in which histamine is released from mast cells, binds to the receptor, and leads to its activation [2]. Because of its special role in hypersensitivity reactions, the histamine H${}_{1}$ receptor is one of the most important targets in the treatment of allergic reactions as well as sleep disorders and vomiting [1,3].

H${}_{1}$R signal transduction is mediated mainly by the G_q_ family. After activation and subsequent H${}_{1}$R-G_q_ dissociation, the Gα subunit can stimulate the phospholipase Cβ, which cleaves the membrane-bound lipid phosphatidylinositol-4,5-bisphosphate (PIP2) into the secondary messengers inositol-1,4,5-trisphosphate (IP3) and diacylglycerol (DAG). While IP3, upon binding to the IP3 receptor, causes the release of secondary messengers such as calcium ions (${\mathrm{Ca}}^{2+}$), DAG, as well as the released ${\mathrm{Ca}}^{2+}$, ensure the stimulation of protein kinase C (PKC) [4,5]. The latter causes the activation of the NF-κB pathway for the immune response signal and thus enables the cell to respond according to the signaled information [6,7,8].

In 2021, the structure of a ternary histamine-H${}_{1}$R-G_q_ complex was published [9], which represented the first experimental structure of histamine in complex with a histamine receptor. This structure allowed the proposal of a “squash to activate and expand to deactivate” mechanism [9]. In this process, the agonist histamine activates the receptor by forming hydrogen bonds with residues in the transmembrane helices H3 and H6 to ”squash” the ligand-binding pocket on the extracellular side while creating a lever-like tension that opens the cavity for the G protein on the intracellular side. In contrast, bulky antagonists expand the orthosteric binding pocket, keeping the transmembrane helices in place and the G-protein binding pocket closed [9]. Despite this important mechanistic information gained from the histamine-H${}_{1}$R-G_q_ ternary complex, one structural detail still remained unclear: since hydrogen atoms are not resolved due to the limited resolution (3.3 Å) of this cryo-EM structure, the protonation state of the bound histamine could not be determined.

**Figure 1:**
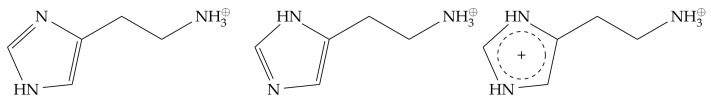
Different histamine protonation forms investigated in this work. Left: monocationic τ-protonated histamine tautomer. Middle: monocationic π-tautomer. Right: dicationic histamine, in which both the τ and the π positions are protonated.

In principle, histamine can exist in two different charge states, either a monocationic or a dicationic form (Figure 1). The monocationic form contains a charged ammonium group in the aliphatic sidechain, while the imidazole ring is singly protonated and consequently uncharged. Depending on the position of the proton, two tautomeric forms exist, referred to as τ-histamine and π-histamine (Figure 1). The τ-tautomer (pK_a_ 6.16) was reported to be a slightly weaker base than the π-tautomer (pK_a_ 6.79) [10]. Whereas most previous modeling studies assumed a monocationic histamine as a ligand [11,12,13], an NMR study by Ratnala et al. [14] has shown that the H_1_R can bind two different charge states of histamine, suggesting that dicationic histamine might also represent a physiologically relevant form. Based on the structure of the histamine-H${}_{1}$R-G_q_ ternary complex, we, therefore, investigated which histamine charge state best stabilizes the active conformation of the receptor. For that purpose, we performed molecular dynamics simulations to investigate the H${}_{1}$R binding properties of different histamine protonation states (i.e., monocationic τ- and π-tautomers as well as the dicationic form).

## 2. Results

### 2.1. Initial Structural Analysis of the Histamine-H${}_{1}$R-G_q_ Complex

**Figure 2:**
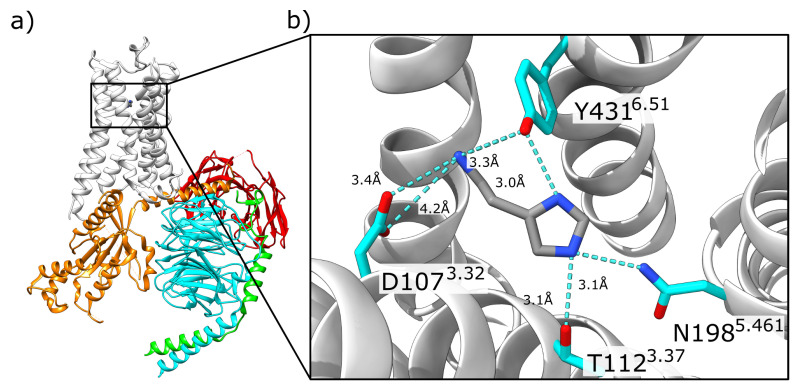
H${}_{1}$R-histamine interaction in the ternary histamine-H${}_{1}$R-G_q_ complex. (**a**) Overall topology of the complex. The receptor is shown as a white ribbon, the Gα in orange, Gβ in cyan, Gγ in green, and a stabilizing antibody in red. (**b**) Enlargement of the histamine binding site. The histamine and interacting residues of the receptor are shown as sticks. Short distances between polar groups are highlighted with dashed lines indicating the distance in Angstrom (Å). The Ballesteros–Weinstein numbering scheme [15] is used to indicate residue position.

The cryo-EM structure of the ternary histamine-H${}_{1}$R-G_q_ complex (PDB: 7DFL) provided the first experimental information about the interactions of histamine in the orthosteric binding pocket. However, due to the limited resolution of 3.3 Å, protons are not visible in the structure. Therefore, we first performed an analysis of the static structure to characterize the polar interactions formed between histamine and the H_1_R (Figure 2). D107^3.32^ acts as an anchor residue, establishing a salt bridge to the charged ammonium group of the histamine. The side chains of residues T112, N198, and Y431, which are in the vicinity of the imidazole nitrogens, can function both as donors and as acceptors of hydrogen bonds. This suggests that each tautomer (i.e., τ- or π-protonated) or even a diprotonated imidazole ring could be accommodated in the binding pocket (Figure 2b).

Since the protonation state of histamine cannot be unambiguously determined on the basis of the static structure, we performed comparative molecular dynamics (MD) simulations of the two monocationic tautomers and the dicationic form of histamine. These simulations are described in Section 2.2 and Section 2.3. In the respective analyses, amino acids are labeled with superscripts according to the Ballesteros–Weinstein (BW) nomenclature [15], which is explained in more detail at the end of the methods section.

### 2.2. Monocationic Histamine Tautomers

**Figure 3:**
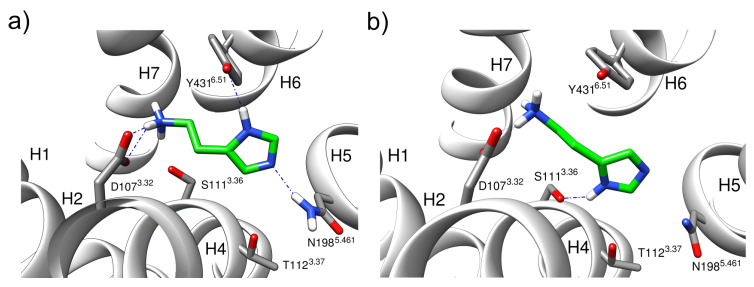
Hydrogen bonding patterns of the ternary histamine H${}_{1}$R-G_q_(α5) complex with the π-histamine tautomer (H${}_{1}$R-HSM-π-α5). (**a**) Initial histamine position. (**b**) Predominant binding mode with a hydrogen bond between the imidazole ring and S111^3.36^. Blue dashed lines indicate distances below 2.5 Å. H1–H7 denote the transmembrane helices of the receptor.

The first set of simulations aimed to study the interaction of histamine tautomers with the active H${}_{1}$R. To ensure maintenance of the active state, the α5-helix of the G_q_-protein was included in the simulations in addition to the H${}_{1}$R. Depending on the histamine (HSM) tautomer investigated, these simulations are hereafter referred to as H${}_{1}$R-HSM-π-α5 or H${}_{1}$R-HSM-τ-α5. In the case of π-protonation, the initial interaction of the histamine imidazole ring with Y431^6.51^ formed in the crystal structure did not remain stable (Figure 3).

**Figure 4:**
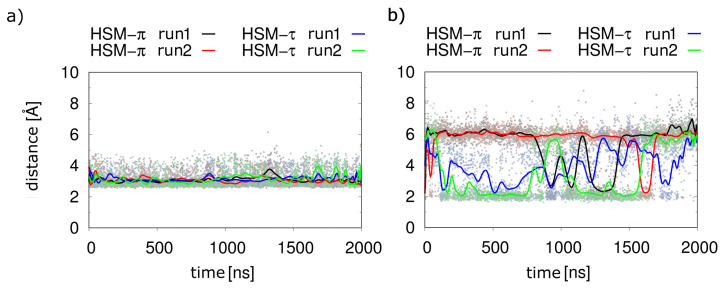
Interactions of the different histamine tautomers. (**a**) Distance between the nitrogen of the histamine ammonium group and the carboxyl oxygens of the D107^3.32^. (**b**) Distance between the π-hydrogen of the imidazole ring and the oxygen of the hydroxy group of Y431^6.51^ for the π-tautomer shown in black and red lines for run1 and run2, respectively. Distance between the π-nitrogen atom of the imidazole ring and the hydrogen of the hydroxy group of the Y431^6.51^ for the τ-tautomer shown in blue and green lines for run1 and run2, respectively. In both representations, explicit values are highlighted by dots, and running averages are shown as lines.

In the first nanoseconds of the simulation, the hydrogen bond to the tyrosine hydroxy group was lost, leading to a rotation of histamine in the binding pocket that affects both the imidazole ring and the aliphatic sidechain. This motion resulted in a rotation of the imidazole ring by about 180°, so that the protonated π-nitrogen interacted with the residue S111^3.36^ (Figure 3b). In contrast, this type of rotation has no effect on the salt bridge of the histamine ammonium group to the strictly conserved D107^3.32^, which remained stable (Figure 4a). This conformation was adopted throughout most of the simulation, with infrequent fluctuations of the imidazole ring allowing recurrent short-lived interactions with Y431^6.51^ (Figure 4b).

**Figure 5:**
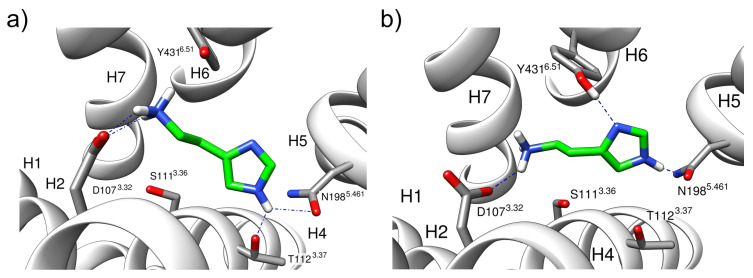
Hydrogen bonding patterns of the ternary histamine-H${}_{1}$R-G_q_(α5) complex with the τ-histamine tautomer (H${}_{1}$R-HSM-τ-α5) (**a**) Hydrogen bonding interaction between N198^5.461^ and T112^3.37^. (**b**) Hydrogen bonding interaction between N198^5.461^ and Y431^6.51^.

In simulations of the τ-tautomer (H${}_{1}$R-HSM-τ-α5), no 180° rotation of the ring was observed, and the histamine adopted in both simulation runs a conformation similar to the experimental binding mode from the cryoEM (Figure 2b). The interactions to N198^5.461^ and T112^3.37^ observed in the experimental structure were preserved in the case of the protonated τ nitrogen, with the interaction with N198^5.461^ preferentially formed in about 90% of all structures. This interaction pattern already emerged at the beginning of the simulation after about 50 ns. The interaction with Y431^6.51^, which was suggested from the crystal structure, was also observed (Figure 2b), but only in the form of transient interactions (Figure 4b) and only when no simultaneous interaction with T112^3.37^ was formed (Figure 5b).

**Figure 6:**
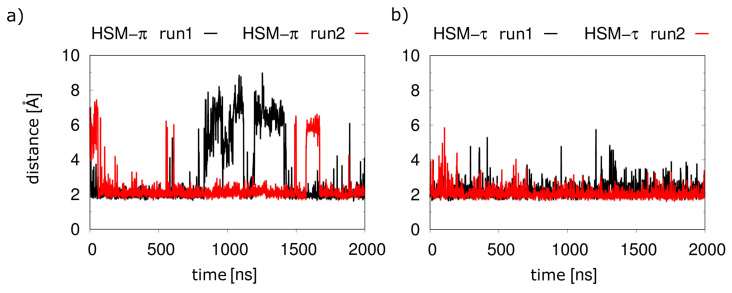
Key hydrogen bonds for different histamine tautomers in the H${}_{1}$R-α5 complex. (**a**) Distance between the hydrogen of the protonated π-nitrogen and the Oγ-oxygen of S111^3.36^. (**b**) Distance between the hydrogen of the protonated τ nitrogen and the Oδ-oxygen of N198^5.461^.

A comparison of the key hydrogen bonds (Figure 6) showed that these were more stable for the τ-tautomer than for the π-tautomer. The distances between the hydrogen of the protonated τ-nitrogen and the oxygen of N198^5.461^ (Figure 6b) were stable in both runs, whereas larger fluctuations occured for the interaction of the π-tautomer with S111^3.36^ (Figure 6a).

It should be noted that the salt bridge of the ammonium group in the aliphatic histamine sidechain to the strongly conserved D107^3.32^ is preserved in all tautomer simulations with G_q_(α5). Despite this common principle, there are essentially two findings that render the τ-tautomer more likely than the π-tautomer for interacting with the active H${}_{1}$R:

(i) The conformation of the τ-histamine fits better to the binding mode from the experimental structure, and no 180° rotational fluctuations of the ring are observed during the simulations as in the π-tautomer.
(ii) The hydrogen bond network of the imidazole ring is more stable for the τ-tautomer (Figure 6).

A second set of simulations was used to investigate the extent to which the G protein stabilizes the histamine interaction. For this purpose, simulations were performed in the complete absence of G_q_.

**Figure 7:**
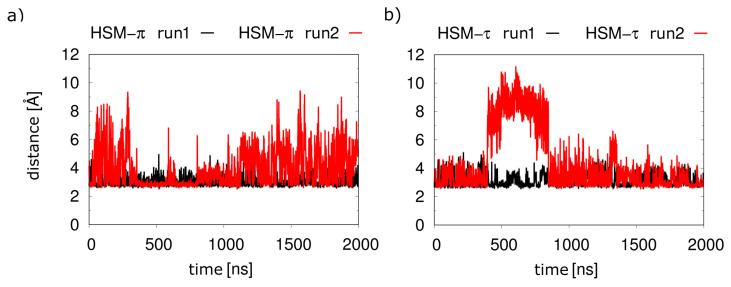
Interaction of histamine with D107^3.32^ in the absence of G_q_(α5). Distances between the ammonium nitrogen of histamine and the carboxyl oxygens of D107^3.32^ for (**a**) the π-tautomer and (**b**) the τ-tautomer.

In the simulations without the G_q_(α5) helix, both histamine tautomers displayed a significantly less stable binding. This is particularly evident from the histamine-D107^3.32^ distance: independently of the tautomer, significant fluctuations of the distance of histamine with its anchor moiety were observed (Figure 7), which suggested a weaker binding compared to the ternary complex investigated above (Figure 4a).

**Figure 8:**
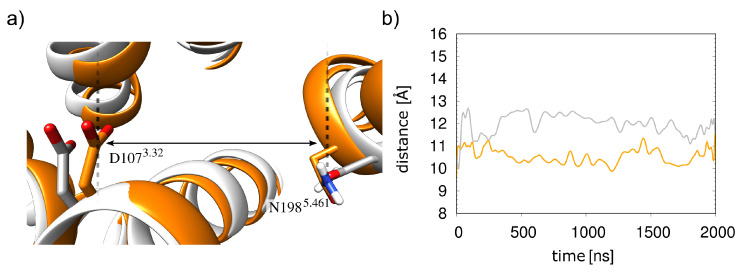
Histamine-binding pocket in the presence and absence of G_q_(α5). (**a**) The structure from the simulations with and without α5-helix are shown in orange and white, respectively. (**b**) Distances between the D107^3.32^ carboxylate and the N198^5.461^ side-chain nitrogen as measured during the simulations, in the same colors as in (**a**).

This increased mobility of the histamine results from an opening of the orthosteric pocket compared to the α5-bound receptor. The opening leads to a weaker interaction with histamine since D107^3.32^ and N198^5.461^, the major interacting residues, are approximately 1 Å farther apart (Figure 8). In summary, the presence of the G_q_ protein thus stabilizes the H${}_{1}$R-histamine interactions.

### 2.3. Dicationic Histamine

The dicationic form of histamine (${\mathrm{HSM}}^{2+}$), whose interaction with the H${}_{1}$R was demonstrated using NMR spectroscopy [14], showed significantly different behavior in the MD simulations than the monocationic form (HSM). While in all simulations of the monocationic state, the presence of the G_q_α5 helix stabilized the interaction, the dicationic histamine dissociated in one of the simulations that included the α5 helix. The other simulation run of this system also showed high flexibility of the histamine.

**Figure 9:**
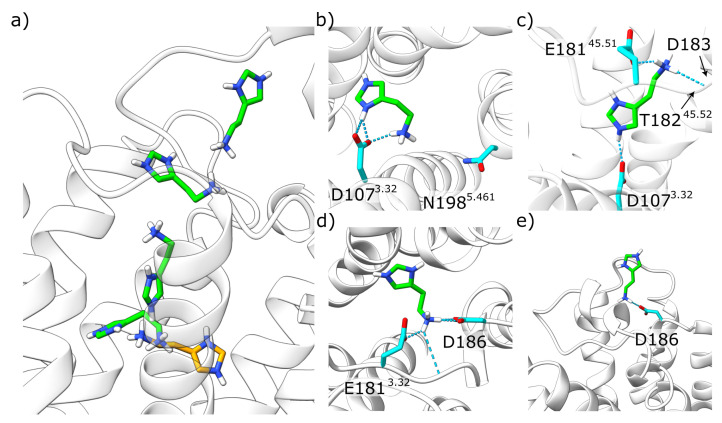
Dissociation of the ${\mathrm{HSM}}^{2+}$. (**a**) Histamine positions observed during dissociation. Histamine is shown by carbon atoms highlighted in green in sticks The starting position from the crystal structure is shown in orange, the receptor in white band representation. (**b**–**e**) show the structures from the H${}_{1}$R-${\mathrm{HSM}}^{2+}$-α5 run 2 at 0 ns, 100 ns, 240 ns, and 346 ns. Interactions are shown by dashed lines.

The molecular origin for this instability can be rationalized with a close examination of the histamine dissociation from the orthosteric pocket (Figure 9). First, the charged imidazole ring shifted from its original binding position and contacted D107^3.32^ (Figure 9b) just as the ammonium group of the histamine side chain did. By increasing the local positive charge density near the negatively charged D107^3.32^, an easier release of the otherwise tightly bound ammonium group of histamine could occur. Therefore, histamine was able to interact with E181^45.51^, which extends into the orthosteric pocket (Figure 9c). Hydrogen bonding to the peptide group between T182^45.52^ and D183 helped stabilize this position (Figure 9c). Subsequently, E181^45.51^ shifted outwards, allowing contact formation with D186 (Figure 9d). This further exposed the ligand to the solvent, allowing complete dissociation to occur shortly thereafter (Figure 9e).

**Figure 10:**
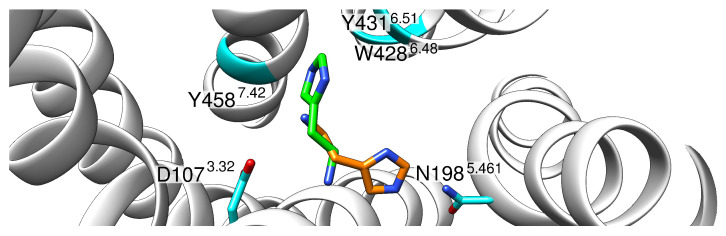
Rearranged binding position of ${\mathrm{HSM}}^{2+}$. Histamine coordinates before (orange) and after (green) rearrangement. Histamine is shown with green/orange carbon atoms, and interacting polar residues are shown in cyan. Positions of the aromatic residues Y458^7.42^, Y431^6.51^, as well as W428^6.48^, are marked while their sidechains are not displayed to enhance clarity.

In the simulations without an α5 helix, ${\mathrm{HSM}}^{2+}$ remained bound in the orthosteric pocket in both simulation runs. In contrast to run2, which only showed increased fluctuations of ${\mathrm{HSM}}^{2+}$ in the orthosteric pocket, a drastic change in binding mode was observed in run1 (Figure 10), which was evaluated in more detail below.

In the newly occupied position, the ammonium group was still tightly bound to D107^3.32^, but the imidazole ring assumed a position rotated by 120° where it was stabilized with interactions with the side chains of residues Y458^7.42^ and Y431^6.51^, as well as W428^6.48^.

The shift in the binding mode of histamine was accompanied by an inward movement of the intracellular side of H6, as is often observed during GPCR inactivation. To further investigate this finding, the distance between the R125^3.50^ and E410^6.30^ residues that form the ionic lock in other receptors was analyzed.

**Figure 11:**
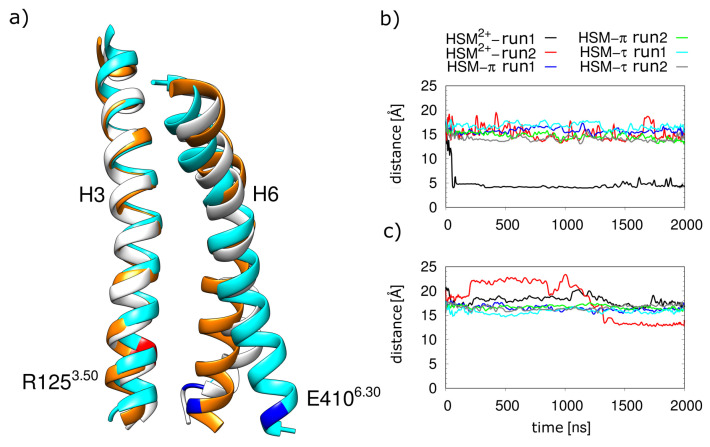
(**a**) Overlay of helices H3 and H6 for the inactive (3RZE [16]; orange) and active (7DFL [9]; cyan) H${}_{1}$R and with a representative structure from simulation run 1 of ${\mathrm{HSM}}^{2+}$ without G_q_(α5). Residues R125 and E410 are marked red and blue, respectively, in the band plot. Distance between R125^3.50^ to E410^6.30^ (**b**) without G_q_(α5) and (**c**) with G_q_(α5).

Analysis of the ionic lock distances revealed a strong decrease and formation of a stable salt bridge in run1 of the α5-free simulation. As shown in Figure 11a, there was an inward movement of H6 that favors the formation of the ionic lock.

Further, for ${\mathrm{HSM}}^{2+}$ (run2), in which no hlionic lock is observed (Figure 11b), stronger fluctuations in the H3-H6 distance are visible than for the monocationic tautomers. Therefore, despite the differences in the exact motion observed, both simulation runs indicate that ${\mathrm{HSM}}^{2+}$ is significantly less compatible with the active H${}_{1}$R conformation than ${\mathrm{HSM}}^{+}$ and might rather represent an interaction partner of the inactive H${}_{1}$R. This conclusion is supported by the observation that even in the presence of the α5 helix, higher fluctuations are observed for the simulations with ${\mathrm{HSM}}^{2+}$ compared to ${\mathrm{HSM}}^{+}$ (Figure 11c).

## 3. Discussion

In solution, histamine can exist in two different tautomers, a π- and a τ-form. In contrast to previous studies on histamine protonation, which were based on quantum mechanical calculations in aqueous solution [17,18], the present study explicitly considered the influence of the receptor. Our MD simulations revealed clear differences between both tautomers with respect to the interaction patterns and the stability of the binding conformations. In the simulations, the π-tautomer initially established a stable interaction pattern with the receptor after minimization and formed polar interactions with D107^3.32^, Y431^6.51^, and N198^5.461^ (Figure 3a). However, in the MD simulation, this mode of interaction proved unstable and resulted in a 180° rotation of the histamine ring in the binding pocket (Figure 3). This caused a loss of contacts with N198^5.461^, and alternative contacts were formed with S111^3.36^ instead. A mutation study by Xia et al., however, showed that mutation of S111 to alanine has little effect on ligand-induced H${}_{1}$R activation, indicating that S111 does not participate in the forming of the hydrogen bond network with the ligand [9]. This finding, together with the 180° rotation of the ring, argues against the physiological relevance of the π-tautomer. In contrast, the preferred conformation of the τ-tautomer is in good agreement with the structure from the cryo-EM and formed stable interactions to N198^5.461^ in the MD-simulations (Figure 5). This finding also agrees well with mutation studies describing the importance of N198^5.461^ for H${}_{1}$R activation [19]. The results show the advantages of molecular dynamics compared to static structural analysis. Based on the crystal structure and static analysis, the differences in conformational stability would not have been apparent. However, one should keep in mind that the initial H${}_{1}$R coordinates used might also affect the relative tautomer stability. Therefore, it cannot completely be ruled out that slight rearrangements of the sidechains in the orthosteric pocket of the active H${}_{1}$R will allow for more favorable interactions of the π-tautomer. This issue is beyond the scope of the present work, but might be addressed by a more complex simulation setup in the future. For example, one could constrain the π-protonated histamine in its initial conformation (thereby preventing 180° rotation) and only relax the H${}_{1}$R conformation using MD simulations. If the H${}_{1}$R remains stable under these conditions, longer MD simulations without histamine restraints would allow verifying the existence of an alternative stable binding mode for the π-tautomer. Another alternative to address this question could be docking studies for different histamine tautomers as ligands, explicitly considering the flexibility of the H${}_{1}$R side chains in the orthosteric pocket.

Another finding of this work was the stabilizing effect of the G protein on the histamine-H${}_{1}$R interaction. When the receptor is stabilized with the G protein, histamine occupies a stable position in the receptor (Figure 4). Without the G protein, larger fluctuations are observed (Figure 7), which can be explained by an opening of the orthosteric binding pocket in the absence of the G protein (Figure 8). This is consistent with the observation that the water-accessible region of the orthosteric binding pocket is larger in inactive GPCRs compared to the active conformation [9,20]. These findings are also in line with previous assumptions regarding the H${}_{1}$R activation mechanism, in which the agonist favors contraction of the orthosteric pocket [9]. However, according to the results of the simulations performed here, histamine alone can only partially stabilize the closed form of the orthosteric binding pocket in the absence of the G_q_ protein.

An NMR study by Ratnala et al. [14] showed that H${}_{1}$R-bound histamine can exist both in a monocationic form and in a dicationic form with an additionally charged imidazole ring. The H${}_{1}$R binding properties of this dicationic form were therefore investigated using MD-simulations analogous to the tautomers described above. The results of these simulations indicate that the active H${}_{1}$R prefers the monocationic form. In particular, this is evident from the higher mobility of the dicationic histamine, which also led to the dissociation of the histamine in the presence of the G protein in one of the simulation runs (Figure 9). Additionally, in both simulations without G protein, the dication induces conformational instability in the active H${}_{1}$R (Figure 11b) and even induced a rearrangement towards an inactive conformation in one of the simulation runs (Figure 10). For better statistical significance of this inactivation motion, more and longer simulation runs would be needed, but nevertheless the already conducted simulations offer first evidence for the compatibility of the dicationic histamine with the inactive H${}_{1}$R (Figure 11). An independent indication of such a role was already found in the study carried out by Ratnala et al., who proposed a protonation-dependent switch for the H${}_{1}$R, analogous to rhodopsin [14]. According to the results of the current study, such a switch could be that dicationic histamine binds to the inactive H${}_{1}$R and then promotes the activation of the H${}_{1}$R by a transition to the monocationic form. However, further studies required to confirm this mechanism are both experimentally and theoretically challenging: experimentally, measurements would be necessary at different pH values corresponding to the different histamine protonation states. However, this is accompanied by the problem of H${}_{1}$R stability, whose structure is also strongly influenced by pH. This makes it difficult to clearly assign observed effects to a change in the histamine protonation state. With simulations, it would first have to be validated that the dicationic histamine was indeed the preferred ligand of the inactive H${}_{1}$R. A verification of the postulated protonation-dependent switch for the H${}_{1}$R [14] would further require simulating a proton transfer from the dicationic histamine to the H${}_{1}$R. This is not possible with conventional MD but will require MD simulations at constant pH to allow proton transfer between the ligand and protein.

In summary, our study was able to establish protonation-dependent differences in the receptor binding properties of histamine and indicate that the monocationic τ-tautomer is the preferred ligand of the active H${}_{1}$R. These differences in the binding behavior were not apparent from the static structural models of the complexes, but could only be captured by simulating the dynamics. This emphasizes the value of MD simulations as a method for the atomistic investigation of protein-ligand complexes and thus also for the development of active substances with tailor-made binding properties.

## 4. Materials and Methods

The coordinates from the PDB entry 7DFL [9] were used as the starting structure for the simulations of the ternary histamine-H_1_R-G_q_ complex with its ligand histamine. Residues 224–401 forming the intracellular loop region (ICL3) between H5 and H6 were deleted from the expression construct for this cryo-EM structure [9]. Xia et al. also demonstrated that the ICL3-deleted receptor still responds to ligand binding, although with increased EC_50_ compared wild-type receptor [9]. According to the AlphaFold-2 model (https://alphafold.ebi.ac.uk/entry/P35367 (accessed on 2 April 2023)), the ICL3 does not contain significant elements of secondary structure and does not adopt a stable 3D fold. Therefore, microsecond MD simulations appear insufficient for an exhaustive conformational sampling of this loop. Such simulations would most probably result in heterogeneous conformations and interactions of the ICL3 that are difficult to interpret due to the lack of proper statistics. For that reason, we have replaced the missing residues 222–404 with a 9-residue GSGSGSGSG-spacer in the system preparation. According to our experience, using a spacer is superior to simulations with unlinked transmembrane helices because the spacer allows for keeping the helix ends in close spatial proximity, as observed in the experimental structures.

Of the G_q_ protein, only the α5 helix (residues 334–359), which represents the major H${}_{1}$R interaction site, was retained in the complex. Six systems were generated that differ by the presence/absence of the G_q_(α5) helix and the histamine protonation state (Table 1). For the lipid environment, a preequilibrated DOPC membrane was used similar to prior publications [21].

**Figure 12:**
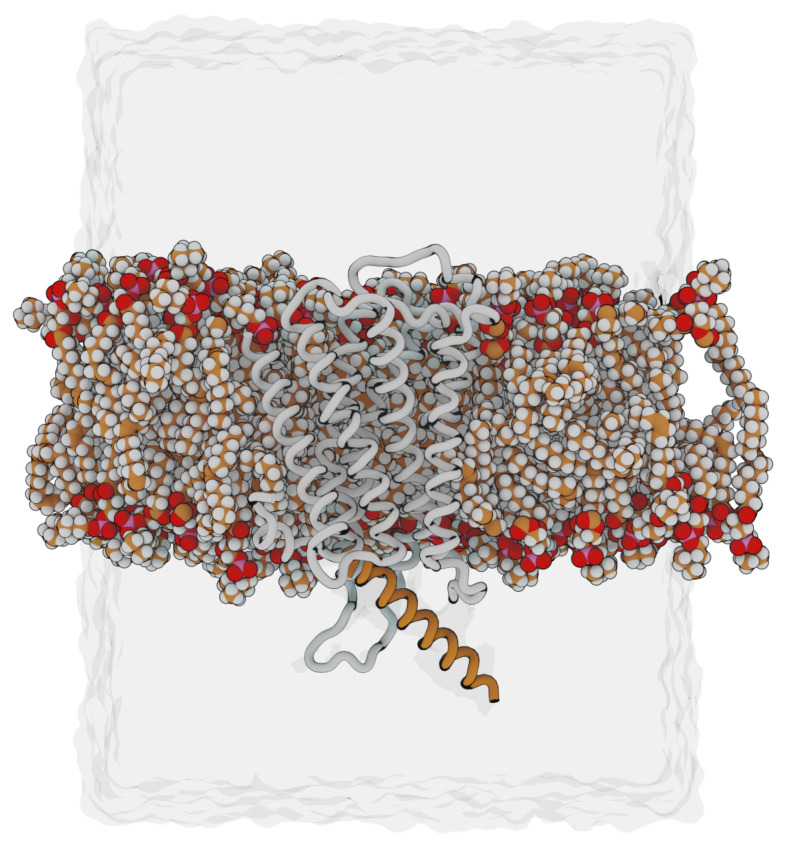
Overview of the simulated system. The H${}_{1}$R and the G_q_ α5-helix are shown as white and orange tubes, respectively. Phospholipids are shown in a space-filled presentation with carbons in orange. Only a subset of the phospholipids is shown to allow for a side-view of the embedded receptor.

All simulations were performed using Amber [22]. The force field implementation FF14SB [23] was used for proteins, while lipid14 [24] was used for DOPC molecules. The relevant histamine forms were parameterized separately following the same steps as in [11,21]. Before calculating RESP atomic charges, a structural optimization was carried out, and a frequency calculation ensured the minimum found. For this, R.E.D. with GAMESS  [25] was used. Missing parameters were automatically assigned using parmchk2 from the AmberTools suite [22]. RESP charges were derived using the R.E.D. server [26]. Water was described via the TIP3P model [27]. The system was neutralized using ${\mathrm{Na}}^{+}$ and ${\mathrm{Cl}}^{-}$ ions for which the Li and Merz 12-6 model was applied [28]. The system was embedded in a simulation box of rectangular shape (Figure 12). Each system was minimized and equilibrated according to a uniform protocol.

The minimization consisted of three successive steps with restraints applied to different subsets of atoms (first to all atoms except water molecules, then to Cα atoms only, and finally without any restraints). During minimization, 2500 steps of the steepest descent algorithm were applied, followed by 2500 steps of the conjugate gradient algorithm. A harmonic potential with a force constant of 10 kcal·mol${}^{-1}$·Å${}^{-2}$ was used for the atomic restraints. Membrane equilibration was performed in 300 consecutive Gromacs [29] simulations of 100 ps each. At this stage, water molecules, diffused into the membrane, were removed while the receptor and ligand atoms were held with a force constant of 5 kcal·mol${}^{-1}$·Å${}^{-2}$. The temperature was kept constant at 310 K using a Berendsen thermostat [30]. Surface tension coupling was applied with a reference pressure of 1 bar and a reference surface tension of 1.1 nm·bar. After membrane equilibration, the systems were converted to an Amber format and underwent a final equilibration cycle analogous to the steps performed previously. The SHAKE algorithm [31] allowed a time step of 2 fs during the equilibration and production runs. Periodic boundary conditions were set for the *x*, *y*, and *z* directions. A summary of all simulation runs performed can be found in Table 1.

Structural analyses were performed with the tool cpptraj from Amber [22]. To allow for a comparison to other GPCRs, H${}_{1}$R residues are labeled by superscripts according to the Ballesteros–Weinstein (BW) nomenclature [15] in addition to their H${}_{1}$R sequence position. In the BW numbering scheme, the most conserved position of each transmembrane helix is assigned the number 50. For example, in H${}_{1}$R, D73^2.50^ indicates that D73 is the most conserved residue of transmembrane helix 2. The other residues in the N- and C-directions are numbered relative to this position (e.g., A72^2.49^ is N-terminally adjacent to D73^2.50^). Residues with three-digit superscripts (e.g., 461 for N198^5.461^) indicate an extension of the original BW nomenclature that was introduced for nonconserved positions by the GPCRdb [32].

## Figures and Tables

**Table 1:** Overview of the simulations performed for the histamine-H${}_{1}$R-G_q_ system. The table describes the composition of the systems, i.e., in which protonation state the ligand histamine or the G_q_-α5 helix (✓) was present. The symbol (×) indicates the absence of the respective component in the arrangement.

System Name	Runs × Time	Histamine	α5 Helix	# Atoms	# DOPC
H${}_{1}$R-HSM-π	2 × 2 μs	π	×	125,354	277
H${}_{1}$R-HSM-π-α5	2 × 2 μs	π	✓	125,794	277
H${}_{1}$R-HSM-τ	2 × 2 μs	τ	×	124,943	278
H${}_{1}$R-HSM-τ-α5	2 × 2 μs	τ	✓	125,392	278
H${}_{1}$R-${\mathrm{HSM}}^{2+}$	2 × 2 μs	$${}^{2+}$$	×	124,942	278
H${}_{1}$R-${\mathrm{HSM}}^{2+}$-α5	2 × 2 μs	$${}^{2+}$$	✓	125,391	278

## Data Availability

Raw data is available upon request.

## References

[1] Hill S.J. (1990). Distribution, properties, and functional characteristics of three classes of histamine receptor. Pharmacol. Rev..

[2] Simons F.E.R. (2004). Advances in H1-antihistamines. N. Engl. J. Med..

[3] Akdis C.A., Simons F.E.R. (2006). Histamine receptors are hot in immunopharmacology. Eur. J. Pharmacol..

[4] Berridge M.J. (1993). Inositol trisphosphate and calcium signalling. Nature.

[5] Jewison T., Su Y., Disfany F.M., Liang Y., Knox C., Maciejewski A., Poelzer J., Huynh J., Zhou Y., Arndt D. (2014). SMPDB 2.0: Big improvements to the Small Molecule Pathway Database. Nucleic Acids Res..

[6] Smit M., Hoffmann M., Timmerman H., Leurs R. (1999). Molecular properties and signalling pathways of the histamine H1 receptor. Clin. Exp. Allergy.

[7] Fernández-Nogueira P., Noguera-Castells A., Fuster G., Recalde-Percaz L., Moragas N., López-Plana A., Enreig E., Jauregui P., Carbó N., Almendro V. (2018). Histamine receptor 1 inhibition enhances antitumor therapeutic responses through extracellular signal-regulated kinase (ERK) activation in breast cancer. Cancer Lett..

[8] Francis H., Glaser S., DeMorrow S., Gaudio E., Ueno Y., Venter J., Dostal D., Onori P., Franchitto A., Marzioni M. (2008). Small mouse cholangiocytes proliferate in response to H1 histamine receptor stimulation by activation of the IP3/CaMK I/CREB pathway. Am. J. Physiol. Cell Physiol..

[9] Xia R., Wang N., Xu Z., Lu Y., Song J., Zhang A., Guo C., He Y. (2021). Cryo-EM structure of the human histamine H1 receptor/Gq complex. Nat. Commun..

[10] Ganellin C. (1973). The tautomer ratio of histamine. J. Pharm. Pharmacol..

[11] Söldner C.A., Horn A.H., Sticht H. (2018). Binding of histamine to the H1 receptor—A molecular dynamics study. J. Mol. Model..

[12] Durant G.J., Ganellin C.R., Parsons M.E. (1975). Chemical differentiation of histamine H1-and H2-receptor agonists. J. Med. Chem..

[13] Panula P., Chazot P.L., Cowart M., Gutzmer R., Leurs R., Liu W.L.S., Stark H., Thurmond R.L., Haas H.L. (2015). International Union of Basic and Clinical Pharmacology. XCVIII. Histamine Receptors. Pharmacol. Rev..

[14] Ratnala V.R., Kiihne S.R., Buda F., Leurs R., de Groot H.J., DeGrip W.J. (2007). Solid-state NMR evidence for a protonation switch in the binding pocket of the H1 receptor upon binding of the agonist histamine. J. Am. Chem. Soc..

[15] Ballesteros J.A., Weinstein H. (1995). Integrated Methods for the Construction of Three-Dimensional Models and Computational Probing of Structure-Function Relations in G Protein-Coupled Receptors. Methods in Neurosciences.

[16] Shimamura T., Shiroishi M., Weyand S., Tsujimoto H., Winter G., Katritch V., Abagyan R., Cherezov V., Liu W., Han G.W. (2011). Structure of the human histamine H1 receptor complex with doxepin. Nature.

[17] Worth G.A., King P.M., Richards W.G. (1990). Histamine tautomerism and its mode of action. Biochim. Biophys. Acta (BBA)—Gen. Subj..

[18] Karpińska G., Dobrowolski J.C., Mazurek A.P. (1996). Tautomerism of histamine revisited. J. Mol. Struct. THEOCHEM.

[19] Bruysters M., Pertz H.H., Teunissen A., Bakker R.A., Gillard M., Chatelain P., Schunack W., Timmerman H., Leurs R. (2004). Mutational analysis of the histamine H1-receptor binding pocket of histaprodifens. Eur. J. Pharmacol..

[20] Warne T., Edwards P.C., Doré A.S., Leslie A.G., Tate C.G. (2019). Molecular basis for high-affinity agonist binding in GPCRs. Science.

[21] Conrad M., Söldner C.A., Miao Y., Sticht H. (2020). Agonist binding and G protein coupling in histamine H2 receptor: A molecular dynamics study. Int. J. Mol. Sci..

[22] Case D.A., Aktulga H.M., Belfon K., Ben-Shalom I., Brozell S.R., Cerutti D.S., Cheatham T.E., Cruzeiro V.W.D., Darden T.A., Duke R.E. (2021). Amber 2021.

[23] Maier J.A., Martinez C., Kasavajhala K., Wickstrom L., Hauser K.E., Simmerling C. (2015). ff14SB: Improving the accuracy of protein side chain and backbone parameters from ff99SB. J. Chem. Theory Comput..

[24] Dickson C.J., Madej B.D., Skjevik Å.A., Betz R.M., Teigen K., Gould I.R., Walker R.C. (2014). Lipid14: The amber lipid force field. J. Chem. Theory Comput..

[25] Schmidt M.W., Baldridge K.K., Boatz J.A., Elbert S.T., Gordon M.S., Jensen J.H., Koseki S., Matsunaga N., Nguyen K.A., Su S. (1993). General atomic and molecular electronic structure system. J. Comput. Chem..

[26] Dupradeau F.Y., Pigache A., Zaffran T., Savineau C., Lelong R., Grivel N., Lelong D., Rosanski W., Cieplak P. (2010). The R.E.D. Tools: Advances in RESP and ESP charge derivation and force field library building. Phys. Chem. Chem. Phys..

[27] Wang J., Wolf R.M., Caldwell J.W., Kollman P.A., Case D.A. (2004). Development and testing of a general amber force field. J. Comput. Chem..

[28] Li P., Song L.F., Merz K.M. (2015). Systematic parameterization of monovalent ions employing the nonbonded model. J. Chem. Theory Comput..

[29] Van Der Spoel D., Lindahl E., Hess B., Groenhof G., Mark A.E., Berendsen H.J. (2005). GROMACS: Fast, flexible, and free. J. Comput. Chem..

[30] Berendsen H.J., Postma J.v., van Gunsteren W.F., DiNola A., Haak J.R. (1984). Molecular dynamics with coupling to an external bath. J. Chem. Phys..

[31] Ryckaert J.P., Ciccotti G., Berendsen H.J. (1977). Numerical integration of the cartesian equations of motion of a system with constraints: Molecular dynamics of n-alkanes. J. Comput. Phys..

[32] Pándy-Szekeres G., Munk C., Tsonkov T.M., Mordalski S., Harpsøe K., Hauser A.S., Bojarski A.J., Gloriam D.E. (2018). GPCRdb in 2018: Adding GPCR structure models and ligands. Nucleic Acids Res..

